# Burden, causes, and treatment approaches of recurrent pregnancy loss: a scoping review

**DOI:** 10.11604/pamj.2025.52.109.46376

**Published:** 2025-11-13

**Authors:** Alex Bosire, Rose Kosgei, David Gathara, Moses Madadi, Alfred Osoti

**Affiliations:** 1 Department of Obstetrics and Gynaecology, University of Nairobi, Nairobi, Kenya,; 2 Health Services Unit, KEMRI-Wellcome Trust Research Programme, Nairobi, Kenya,; 3 MARCH Centre, London School of Hygiene and Tropical Medicine, London, United Kingdom

**Keywords:** Recurrent pregnancy loss, etiology, management, diagnostic approaches, immunological causes

## Abstract

Recurrent pregnancy loss (RPL), defined as the loss of two or more clinically recognized pregnancies before 20 weeks of gestation, poses a significant global medical challenge. Affecting up to 5% of couples trying to conceive, its multifactorial causes include genetic, anatomical, immunological, hormonal, and environmental factors. However, 65% of cases remain idiopathic, exacerbating the complexity of diagnosis and treatment. Low- and middle-income countries (LMICs) face additional challenges due to limited diagnostic capacities, socioeconomic disparities, and the burden of infectious diseases. Advancements in diagnostic and treatment technologies offer promise but are often inaccessible in resource-limited settings. This scoping review involved systematic searches in PubMed, Cochrane Library, and other sources for studies published since 2014. Eligible studies focused on the aetiology, diagnosis, and treatment of RPL. A total of 61 studies were included, primarily from high-income countries, with gaps noted in data from LMICs. Findings highlight RPL's multifaceted aetiology, including chromosomal abnormalities, uterine anomalies, thrombophilias, infections, and lifestyle factors. Advanced diagnostic methods such as next-generation sequencing enable personalized management but remain inaccessible in most LMICs. Management strategies range from immunotherapy and anticoagulants to surgical interventions and lifestyle modifications. Despite promising advancements, gaps in evidence and resource disparities persist, particularly in LMICs, where cultural stigmas and limited healthcare access further complicate care delivery. Addressing RPL requires a multidisciplinary approach encompassing diagnostics, treatment, and psychological support. Tailored strategies are essential for LMICs where inadequate healthcare infrastructure and socioeconomic barriers hinder progress. Prioritizing research, equitable access, and collaborative global efforts are vital to improving outcomes for affected couples worldwide.

## Introduction

Recurrent pregnancy loss is defined as the failure of two or more clinically recognized pregnancies before 20 weeks of gestation [1] and includes embryonic and foetal losses. Recurrent pregnancy loss poses a significant medical challenge globally, including in low- and middle-income countries (LMICs). Although sporadic pregnancy loss is common, occurring in 15% of pregnancies, RPL impacts approximately 5% of couples trying to conceive [2-5]. Recurrent pregnancy loss is an important reproductive health issue worldwide and is commonly considered one of the most challenging aspects in the field of reproductive medicine, since more than 50% of couples with RPL have no clear etiological explanation.

Recurrent pregnancy loss can be caused by various factors, including chromosomal abnormalities, uterine abnormalities, hormonal imbalances, autoimmune disorders, thrombophilic disorders, infections, advanced maternal age, obesity, environmental factors, and lifestyle factors [6-13]. Maternal factors such as smoking, excessive alcohol consumption, and poor nutrition can also contribute to RPL. Additionally, paternal factors like abnormal sperm parameters or genetic abnormalities may play a role in recurrent miscarriages [14-19]. Consequently, in many cases, RPL may be multifactorial, involving a combination of genetic, anatomical, hormonal, immunological, and environmental factors. While there are several known causes, a considerable proportion - up to 65% - of the RPL causes are idiopathic [20-25]. These idiopathic cases lead to emotional and physical concerns but also contribute to challenging treatment approaches.

The treatment of RPL involves identifying and addressing underlying causes, if possible, and supporting subsequent pregnancies to reduce the risk of further losses [26-31]. Medical evaluation of both partners is necessary to identify any underlying factors contributing to RPL. This evaluation may include genetic testing, hormonal assessments, imaging studies to evaluate uterine structure, and tests for autoimmune disorders and blood-clotting disorders. Treatment may involve addressing specific underlying conditions such as hormonal imbalances, uterine abnormalities, autoimmune disorders, or thrombophilic disorders. For example, hormone therapy, surgery to correct uterine abnormalities, or medications to manage autoimmune or clotting disorders may be recommended. Additionally, genetic counseling and testing, lifestyle modification, assisted reproductive technologies (such as in vitro fertilization), emotional support and counseling, and management of antenatal complications are important approaches in treating RPL. However, the high cost of diagnostic tests and procedures associated with identifying individual causes of RPL may pose challenges in accessing healthcare for patients [32-34].

While the underlying causes of RPL are often multifaceted and frequently remain undetermined, several factors specific to low-middle-income countries (LMICs) contribute to the occurrence and management of RPL. Limited access to comprehensive reproductive healthcare services, including healthcare facilities, can result in delayed diagnosis and treatment. In addition, the scarcity of diagnostic resources, such as expensive and sometimes inaccessible tests, exacerbates the problem. Genetic and maternal immune dysregulation testing is restricted, often only available in the private sector, and unaffordable for many. In LMICs, nutritional factors such as malnutrition and nutrient deficiencies are more prevalent, increasing the risk of pregnancy loss. Infectious diseases like malaria, HIV/AIDS, and sexually transmitted infections (STIs) are more common in LMICs, contributing to pregnancy complications and miscarriage. Moreover, socioeconomic factors like poverty, inadequate education, and limited access to contraception can also contribute to higher fertility rates and subsequent pregnancy losses. Cultural and societal attitudes towards reproductive health and family planning can also influence pregnancy outcomes. Together, these factors hinder prompt diagnosis, management, and support for women with RPL, resulting in suboptimal reporting, burden, and underinvestment in this area. Recent advancements in next-generation sequencing technology have enabled the identification of chromosomal aberrations and single gene variants associated with RPL, shedding light on its pathogenic mechanisms. These advancements facilitate personalized diagnostic evaluations and etiologic diagnoses with targeted therapeutic recommendations, which have shown effectiveness through targeted management. Although these services are not typically available in the public sector in most LMICs, there is potential to leverage private sector capacity and data to advocate for similar investments in the public sector.

Recurrent pregnancy loss is increasingly recognized as a multifactorial disease, and its causation has been attributed to various factors, among which genetic factors and maternal immune dysregulation are considered to be two of the most important causes. However, due to limited diagnostic capacity, restricted access to laboratory services, and the high costs associated with these tests, RPL secondary to these factors in low- and middle-income countries is inadequately documented. This review aims to synthesize existing evidence on the burden of RPL, describe the key risk factors associated with RPL, and, within thematically distinct sections, explore the role of autoimmune and genetic factors and their relationship with autoimmune markers.

## Methods

The reporting of this scoping review follows the Preferred Reporting Items for Systematic Reviews and Meta-Analyses Extension for Scoping Reviews (PRISMA-ScR) guidelines [35].

**Search strategy:** we conducted literature searches in PubMed and Cochrane Library, with additional citation mining from Google Scholar and references from other reviews. The search was limited to papers published from 2014 and written or translated into English. The literature search was limited to studies published or translated into English to ensure accuracy in data extraction and interpretation. While this restriction may introduce potential language bias, English is the predominant language of publication for biomedical research, particularly in journals indexed in PubMed and Cochrane Library. To mitigate the risk of omitting relevant evidence, additional efforts, such as citation tracking from included studies and reference lists, were conducted, which likely captured key studies published in other languages. The search strategy and results from the databases are listed in Table 1. These two databases were selected as primary because they provide the most comprehensive and peer-reviewed coverage of biomedical and clinical studies relevant to reproductive health. To ensure inclusivity, citation mining from Google Scholar and manual reference tracking from related reviews were also conducted to capture potentially missed articles and grey literature. While databases such as Embase or Scopus were not included due to access limitations and overlap in indexed journals with PubMed, the combination of these sources ensured that the search was both systematic and sufficiently broad to minimize bias and omission of key studies.

**Table 1:** the search strategy

PICO (T)	Term	Search terms	String
P	(Pregnant) women of childbearing age; women experiencing RPL	Women, pregnant women, women of childbearing age, pregnancy, RPL, habitual abortion, repeated miscarriage	(“Women” OR “pregnant women” OR “women of childbearing age” OR “pregnancy”) AND (“RPL” OR “habitual abortion” OR “repeated miscarriage”)
			AND
E/I	Various treatment approaches for RPL	Treatment, management, intervention, pharmacological treatment, medication, life	(“RPL” OR “habitual abortion” OR “repeated miscarriage”) AND (“treatment” OR “management” OR “intervention”) OR (“RPL” OR “habitual abortion” OR “repeated miscarriage”) AND (“medication” OR “surgery” OR “life”
			AND/OR
Comparator	Comparisons between different treatment modalities	Comparative effectiveness, randomized controlled trial (RCT), standard care, placebo, treatment modalities	(“RPL” OR “habitual abortion” OR “repeated miscarriage”) AND (“effectiveness” OR “comparative effectiveness” OR “treatment modalities” OR “standard care” OR “randomized controlled trial” OR “RCT” OR “multicentre study”)
			AND/OR
Outcomes	Burden of RPL	Burden, prevalence, incidence, causes, aetiology, risk factors, pregnancy outcome, psychological impact, socioeconomic implications, foetal outcome	(RPL OR habitual abortion OR repeated miscarriage) AND (burden OR prevalence OR incidence OR causes OR aetiology OR risk factors OR pregnancy outcome OR “psychological impact” OR “socioeconomic effect” OR “foetal outcome”)
Time	2014 onwards		

RPL: recurrent pregnancy loss

**Study selection and screening:** two reviewers (AB and DG) jointly discussed and built the search strategy. Afterward, they conducted the searches, after which the retrieved results were screened for relevance, initially by title and abstract as per the search strategy. Using Rayyan [36]. The records eligible by abstract were pushed to full text screening and reviewed against the inclusion criteria by the reviewers. The EndNote reference manager [37] was then used to manage the retrieved papers for easy screening and abstraction, and reasons for ineligibility or exclusion were documented at each stage. Inconsistencies, risk of bias, and disagreements on the eligibility of papers were jointly discussed by the reviewers, plus a third reviewer, RK, at both the abstract and full-text review stages. Studies were considered potentially eligible for inclusion if they reported on the aetiology, diagnosis, or treatment of RPL, were published from 2014, and were published or translated into English.

To improve transparency and reproducibility, eligibility was further refined using the PICOS framework. The review focused on studies involving women of reproductive age diagnosed with RPL, defined as two or more consecutive pregnancy losses. Eligible studies were those that examined autoimmune, genetic, or other etiological factors contributing to RPL, as well as diagnostic and therapeutic approaches addressing the condition. Where applicable, comparisons were made between affected and unaffected populations or between different diagnostic and treatment modalities. The primary outcomes of interest included the identification of etiological factors, diagnostic markers, and reported therapeutic outcomes related to RPL. The review considered a range of study designs, including observational studies (cohort, case-control, and cross-sectional), clinical trials, systematic reviews, and meta-analyses published within the defined review period.

**Outcomes and data extraction:** the reviewers jointly extracted the following information onto a pre-developed MS Excel template from the studies included in the final synthesis: study bibliographic details (author, year of publication, study design, country/region and World Bank classification), type of participants and sample size, terms and concepts used for RPL, causes or risk factors for RPL, and treatment or management interventions. To ensure the credibility and robustness of the findings, a quality appraisal and risk of bias assessment were conducted for all included studies, using tools appropriate to each study design. Observational studies (cohort, case-control, and cross-sectional) were evaluated using the NOS to assess selection, comparability, and outcome domains. Randomized and non-randomized intervention studies were appraised using the RoB 2.0 tool, focusing on sequence generation, allocation concealment, blinding, incomplete outcome data, and selective reporting. For systematic reviews and meta-analyses included in the synthesis, the GRADE approach was applied to determine the overall quality and strength of the body of evidence. Discrepancies between reviewers were resolved through discussion or consultation with a third reviewer (RK). This process ensured that only studies meeting acceptable methodological standards contributed to the evidence synthesis and subsequent meta-analysis.

## Results

**Figure 1:**
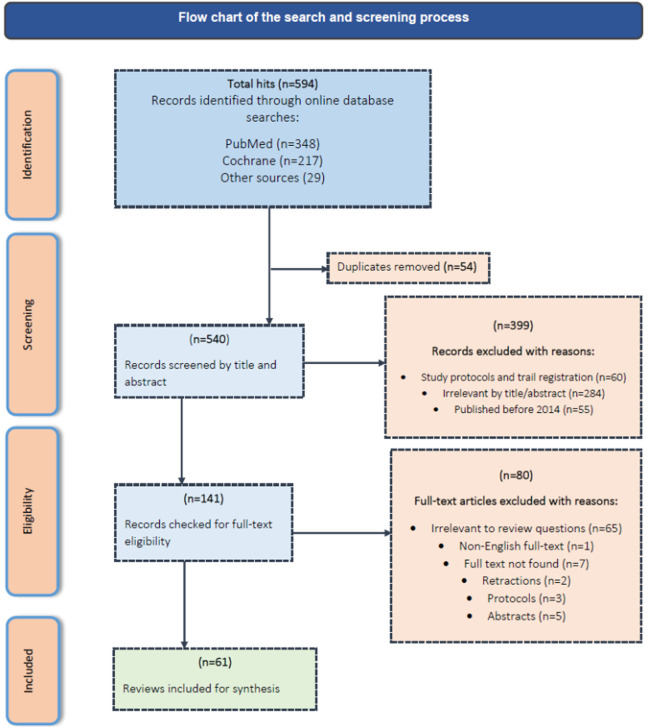
the PRISMA flowchart of the search and screening process

**Study screening and selection:** the PubMed search produced 348 articles, while the search from Cochrane Library yielded 217. Twenty-nine (29) more articles were retrieved from additional manual searches. In total, 594 articles were identified across all searches. Fifty-four (54) articles were removed for duplication, and of the remaining 540 articles, 399 were dropped at title-abstract screening, and a further 80 were removed at full-text screening. Sixty-one (61) records qualified for data charting and synthesis. The summary of the search process is illustrated in Figure 1.

**Study characteristics:** most studies included were reviews (scoping reviews, narrative reviews, systematic reviews, and meta-analyses), 25(41%), classified herein by region as “global” 25(41%). Experimental trials (randomised controlled trials and quasi-experimental studies) accounted for a total of 34.4% (n=21). Of the articles included in this review, none were from sub-Saharan Africa, and only 9(14.8%) were from the Middle East and North Africa. Most (68.9% (n=42) of the included 61 studies were published from 2017. The reported findings below are therefore a reflection of mostly high-income and global contexts (Table 2).

**Table 2:** characteristics of the included studies

Study designs	Number of papers, n (%)
Observational	15(24.6)
Quasi-experimental	4(6.6)
**randomized controlled trial (**RCT)	17(27.9)
Reviews	25(41)
**Publication period**	
2014-2016	19(31.1)
2017-2019	19(31.1)
2020-2022	16(26.2)
2023-2024	7(11.5)
**Sources by World Bank income group classification**	
Lower-middle income	8(13.1)
Upper-middle income	3(4.9)
High income	25(41)
Mixed	25(41)
**Sources by region**	
North America	6(9.8)
Global	25(41)
Middle East and North Africa	9(14.8)
Europe and Central Asia	8(13.1)
East Asia and the Pacific	12(19.7)
South Asia	(11.6)

### Factors associated with the aetiology of recurrent pregnancy loss

**Immunological factors:** immunological disturbances have been suggested to play an important role in RPL in most studies included in this review [2,22,23,38-58]. Maternal immune dysregulation has been observed as one of the leading causes of RPL [38,59,60]. The dysregulation of the maternal immune system typically entails two abnormal responses: 1) lack of activation with negative signals into maternal-foetal tolerance and impaired embryo implantation or placentation, and 2) overactivity with an increased inflammatory uterine environment and damage to the trophoblast [38].

Alongside human leukocyte antigen (HLA) and cytokine allelic variations, elevated Natural Killer (NK) [42,44,46,57,61] cell and T cell expression pattern changes in the endometrium have been implicated in patients with RPL. The NK cells are innate lymphocytes whose activity involves direct cytotoxicity and cytokine production and are important effectors in the killing of target cells. The NK cells express a series of NK receptors (NKRs) from three families - immunoglobulin-like transcripts (ILTs), killer immunoglobulin-like receptors (KIR), and CD94/NKGs - whose combinations coexist to regulate the activity of NK cells by the balance between activating and inhibitory signals transduced by the receptors in the membrane [38,61]. As placentation is regulated by interactions between maternal KIRs expressed by uterine NK cells and foetal HLA-C molecules expressed by extravillous trophoblast (EVT) cells, it is related that insufficient invasion of the uterine lining by the trophoblast and vascular conversion of the decidua are thought to be the primary defects encountered in RPL and other primary defects encountered in preeclampsia foetal growth restriction [38,59,62].

Additionally, conditions such as autoimmune connective tissue diseases, including antiphospholipid antibody syndrome, rheumatoid arthritis, systemic lupus erythematosus, and systemic sclerosis (primary Sjogren´s syndrome and inflammatory myositis) [22,38,39,41,45-47,49,52,53,56,58,59,63-65] can increase the risk of miscarriage by affecting blood flow to the placenta or causing inflammation within the uterus, thereby exerting detrimental effects on trophoblasts.

**Parental chromosomal abnormalities:** recurrent pregnancy loss may be attributed to chromosomal abnormalities in one or both parents [2,3,23,39-41,43,45,53,55,56,59,65-69], which can increase the likelihood of producing embryos with genetic defects by disrupting normal foetal development, impairing implantation, and causing placental dysfunction. Balanced translocation has been regarded as the most frequent chromosomal abnormality, alongside inversions or other structural rearrangements of chromosomes that may disrupt normal embryonic development and lead to RPL. These chromosomal abnormalities mostly originate from random errors in maternal meiosis I, possibly from abnormal segregation during oocyte development, leading to embryos that are non-viable for sustained development [59]. Maternal chromosomal mosaicism, that is, the existence of genetically different cells within an individual, also occurs during embryonic development as a result of chromosomal segregation in the course of mitotic cell division and may contribute to embryonic chromosomal aneuploidy, in addition to random errors in the first meiotic division of the oocyte and parental chromosomal aneuploidy [39,59].

**Thrombophilias:** the term thrombophilia describes a group of disorders that result in an increased risk of abnormal coagulation and, consequently, of venous and arterial thromboembolism. Thrombophilias - inherited or acquired - have been implicated in RPL [23,38-41,43-46,52,56,58,70-73]. Inherited thrombophilias are caused by genetic mutations in genes encoding or regulating common clotting factors. These conditions can impair the normal functioning of the placenta or increase the risk of thrombosis within the uterine vasculature, leading to a hypercoagulable state, compromising foetal blood supply. Thrombosis of spiral arteries within the placenta may affect perfusion and lead to late foetal loss [46]. Pregnancy itself is a prothrombotic state, and therefore pregnant women are at even higher risk for complications when a thrombophilia is also present [38].

**Uterine abnormalities:** many studies have identified the contribution of structural anomalies of the uterus in RPL [2,22,23,39-41,43-47,52,53,55,56,68,72,74-78]. These include congenital uterine malformations such as a septate uterus and a bicornuate uterus, with the septate uterus being the most common uterine anomaly, which may be attributed to different pregnancy disorders, such as first-trimester miscarriage [78,79]. Intrauterine adhesions, also known as Asherman's syndrome, often result after several curettages of gravid or non-gravid uterus infections or surgery, and can impede the implantation success by preventing embryo attachment to the intrauterine luminal surface [78]. In some cases, submucous fibroids and endometrial polyps can expand into the uterine cavity, thereby severely affecting implantation through several mechanisms, including increased uterine contractility, endometrial inflammation, and abnormal vascularization, leading to implantation failure [78]. These abnormalities may interfere with implantation, placental development, or proper foetal growth. In some cases, the mechanisms of recurrent implantation failure include decreased endometrial receptivity, which could be related to both uterine anomalies and endometriosis [39,41,72]. Assessment of the uterine cavity for congenital and acquired anomalies is commonly performed using 3D ultrasonography, saline-infused sonography, MRI, or hysteroscopy [41].

**Endocrinological factors (hormonal imbalances):** hormonal imbalances, particularly those involving the thyroid hormones progesterone, estrogen, and insulin, can disrupt the delicate processes necessary for maintaining a healthy pregnancy [2,3,22,23,40,41,43-46,52,53,55,56,74,80,81]. These imbalances and resultant conditions, such as polycystic ovarian syndrome [41,56,74], diabetes and hyperprolactinemia [23,44,56], and abnormal thyroid function [23,41,44-46,56,80,81], have therefore been associated with poor obstetric outcomes, including RPL. Hyperprolactinemia, for instance, can disrupt ovulation and the hormonal equilibrium necessary for a healthy pregnancy. Additionally, it may lead to irregular menstrual cycles, potentially resulting in RPL. Diabetes that is not properly managed can lead to RPL through a variety of means, such as hyperglycaemia, hormone imbalances, vascular issues, immune system modifications, increased oxidative stress, and a heightened risk of thrombophilia. Some evidence indicates that euthyroid patients with autoimmune thyroid disease (positive thyroid antibodies in the setting of normal TSH and T4 levels) have a higher risk of miscarriage and RPL [46]. In addition, insufficient progesterone levels, as in the case of luteal phase deficiency [44,47], for example, may lead to inadequate endometrial development, making it difficult for the embryo to implant or sustain growth. As a diagnostic approach, thyroid function testing and screening for the presence of thyroid-stimulating hormone and thyroid peroxidase antibodies are recommended in all women with RPL. However, measurement of serum prolactin is recommended in women with RPL unless they have clinical symptoms of hyperprolactinaemia [41].

**Infections:** reproductive tract infections are associated with an increased risk of RPL [41,44,53,55,56,82,83]. Infections can have an impact on the uterine environment, which can lead to complications with implantation and placental development. Chronic endometritis, an infection of the uterine lining, can result from infections with Chlamydia, *Ureaplasma urealyticum*, and *Mycoplasma hominis*. This type of chronic inflammation can interfere with the uterine lining's receptivity, making it less suitable for embryo implantation and increasing the likelihood of miscarriage. Other selected organisms, such as *Listeria monocytogenes, Toxoplasma gondii*, rubella, cytomegalovirus, and herpes virus have been identified in POC samples after spontaneous miscarriages [44]. Infections can have a direct impact on the developing embryo or foetus, which may result in foetal demise or miscarriage. These include cytomegalovirus (CMV), toxoplasmosis, rubella, herpes simplex virus (HSV), and parvovirus B19, all of which have been shown to cause harm during pregnancy. These pathogens can pass through the placental barrier and disrupt foetal development, leading to congenital abnormalities, restricted foetal growth, or foetal death. Infections can also prompt abnormal immune reactions, such as the creation of autoantibodies or activate immune cells that contribute to RPL. These immune responses can result in heightened cytotoxic activity or disrupt the typical immune tolerance mechanisms that are essential for maintaining a healthy pregnancy.

**Male partner factors:** male factors, including higher levels of sperm DNA fragmentation, abortive apoptosis, defective chromatin maturation, and oxidative stress (OS), are the principal mechanisms involved in male infertility and have been associated with the incidence of RPL [40,41,44,52,59,69,84]. However, some antagonistic evidence has reported a limited association between sperm quality and RPL [85].

**Maternal age:** advanced maternal age, that is, 35 years and over, has been linked to an increased risk of RPL [23,39,59,65,86,87]. It has been shown that this association is possibly due to the corresponding increase in frequency of aneuploidy, chromosomal abnormalities, as well as the lower quality of the embryos, including those that are used for in vitro fertilization (IVF). Moreover, it is possible that at advanced maternal ages, there may be higher rates of underlying medical conditions that would impact pregnancy negatively.

**Obesity:** increased BMI (> 25 kg/m^2^) has also been shown to impact implantation rate [2,39,58,69]. As the BMI approaches 30 kg/m^2^ or higher, there has emerged an association with a higher risk of chronic inflammation (relatedly, in the reproductive tract), hormonal disturbances, and insulin resistance; the latter two of which further compound the predisposition to endocrine diseases, including diabetes and PCOS, which further increase the risk of RPL.

**Lifestyle factors:** lifestyle factors like smoking, poor diet, and alcohol intake [2,39,40,45,69]. These lifestyle choices can adversely affect fertility, implantation, and the development of a healthy placenta, thereby predisposing women to miscarriage. Smoking, for instance, has been linked with decreased ovarian function, increased oxidative state (as well as in the male partner), and impaired implantation of the embryo. Poor diet can also result in dietary deficiencies, including folate. Moreover, alcohol intake can also increase the risk of hormonal imbalances, further interfering with implantation and development of the embryo. In addition, improper lifestyle habits may be a predisposing factor to obesity, whose contribution to RPL has been described above.

### Management of recurrent pregnancy loss

**Empiric therapies**

**Immunotherapy:** since the parental immune system has been found to play a significant role in RPL, immune tests that may prove useful in patients with RPL include the following: KIR and HLA-C typing, and an autoimmunity panel: clinically individualized immune tests for metabolic issues or specific autoimmune disorders in a multidisciplinary specialized unit [38,63,64]. As the aetiology of RPL is multifactorial, interdisciplinary collaboration between clinicians, geneticists, and immunologists is essential in the effort to address RPL [58]. The efficacy of immunosuppressive agents such as corticosteroids, intravenous immunoglobulin (IVIG), or tumor necrosis factor-alpha (TNF-α) inhibitors remains controversial as a consideration for women with suspected immune-mediated RPL [39,41,45,46,48,50,54,55,57,74,88-90]. Some RCTs have established to some degree that intravenous immunoglobulins, 400 mg/kg per treatment, every 3 to 4 weeks, could be effective for women with immunologic abortion and documented abnormally elevated NK cells by the downregulation of NK subsets and cytotoxicity [48,54,55,61,74,88,89,91]. However, several reviews and meta-analyses have found no beneficial effect of the use of intravenous immunoglobulins, besides being expensive and with potential serious side effects [41,45-47,72]. Meng *et al*. found that since high NK activity was associated with unexplained recurrent spontaneous abortion (URSA), intralipid and IVIG could equally lower NK activity and improve pregnancy outcomes in patients with URSA, and recommended the use of intralipid as an alternative treatment to IVIG for URSA [57]. As the use of artificial intelligence (AI) has been progressive in recent years, some attempts have been made towards developing predictive models for RPL by the use of an endometrial immunology panel in biochemical pregnancy prediction, since it has been reported that implantation failure in ART is thought to be mainly due to impaired endometrial receptivity [92].

**Genetic screening and matching:** chromosomal analysis is also a necessary component of etiologic research in couples with recurrent miscarriages [67]. As evidence suggests that patients with recurrent implantation failure have more chromosome abnormalities within their embryos, pre-implantation genetic diagnosis (PGD) may be used to evaluate specific chromosomal diseases, especially among IVF patients with specific risk factors [39]. With technological advancement and the cost of whole-genome sequencing decreasing, the technology has become more widely used and clinically applicable [40]. Parental karyotyping has been recommended in cases of women suffering from recurrent implantation failure, but only in specific subgroups such as nulliparous women with a history of miscarriage [39], as well as in men with infertility. As an approach to manage genetic disorders, next-generation sequencing (NGS), and particularly exome sequencing, has been used to identify the gene variants underlying human genetic diseases [59].

**Managing thrombophilias:** the use of antithrombotic agents, often in combination with aspirin and occasionally with prednisolone, is common in women with thrombophilic disorders or antiphospholipid syndrome (APS) and is thought to improve uteroplacental blood flow and reduce the risk of thrombosis. Some studies have reported the beneficial effect of the use of low-molecular-weight heparins (LMWH). However, overall [39,45,52,71,73,93]. This review found no significant evidence reduction in risk of pregnancy loss from the use of LMWH [49,53,70].

### Managing uterine abnormalities

**Surgical interventions:** surgical interventions aim to correct anatomical abnormalities or uterine pathologies that may contribute to RPL [39,41,44,46,76,77]. In the case of a septate uterus, hysteroscopic septum resection of the uterine septum or other intrauterine abnormalities can improve pregnancy outcomes by providing a more favourable environment for implantation and foetal growth [39,41,44] following a uterine evaluation [46]. Surgical removal of uterine fibroids, particularly submucosal or intramural fibroids distorting the uterine cavity, may be indicated in women with RPL associated with fibroid-related complications. Findings by Al-Husban *et al*. however, showed that hysteroscopic myomectomy, compared with hysteroscopic septoplasty, had a higher spontaneous pregnancy rate, more term pregnancies, and lower miscarriage rates [77]. In addition, salpingectomy and, in some cases, tubal occlusion procedures in the presence of hydrosalpinx have been shown to increase the likelihood of implantation success in future IVF cycles [39].

**Intrauterine peripheral blood mononuclear cells (PBMC) therapy:** as it has been discussed, implantation is an essential phenomenon for pregnancy and IVF success. Peripheral blood mononuclear cells (PBMCs) - consisting of B lymphocytes, T lymphocytes, and monocytes - have an important role in immune modulation during pregnancy. Considering the importance of the role of immune cells in pregnancy, scientists have used peripheral blood mononuclear cells (PBMCs) to trigger the initial inflammation [39,78]. This entails isolation of PBMCs from the patient´s peripheral blood, then their intrauterine infusion of the cells during the implantation window. These cells produce many cytokines that have been known to improve endometrial receptivity during implantation. PBMC therapy as a treatment procedure aims at stimulating the necessary initial inflammation for implantation and has demonstrated satisfactory outcomes. In essence, PBMC therapy aims to restore an immune balance and promote maternal-foetal tolerance through immunomodulation, as these cells exert both immunoregulatory and immunosuppressive properties, promoting a tolerant environment for the pregnancy. This treatment approach holds great potential, although there is still a need for more research to optimize the treatment protocol - dosage, timing, and frequency of infusions.

### Other treatment approaches

**Optimal in vitro fertilization treatment:** optimal IVF treatment with embryo transfer allows for controlled ovarian stimulation, in vitro fertilization of oocytes, and selection of high-quality embryos for transfer, bypassing potential barriers to implantation and early pregnancy loss. This may be achieved through an ovulation induction protocol, progesterone support, and ultrasound-guided transfer of a quality embryo [39]. In addition, it has been demonstrated that infusion of platelet-rich plasma (PRP) into the uterus of patients with RPL before embryo transfer in the ICSI cycle could increase the chance of live birth, although this effect was not statistically significant [94]. Platelet-rich plasma contains growth factors and cytokines that may promote endometrial regeneration and enhance embryo implantation in women with refractory RPL.

**Treating infections:** addressing implicated infections of the reproductive tract with tailored antibiotic therapy can help eradicate offending organisms and thereby create a healthy uterine environment for implantation. Chronic endometritis (CE) is a uterine pathology that has been associated with RPL. Traditionally, CE has been diagnosed on histological examination, on visualization with hysteroscopy, and by bacterial culture [39]. Evidence demonstrates that treatment of chronic endometritis alongside other uterine infections with antibiotics significantly improves the pregnancy outcomes in women with RPL, leading to more successful implantation rates in future cycles [39,41,44,56,82,83]. In addition, regular follow-up and monitoring would likely be essential for lowering the risk of recurrences.

**Managing male factor:** for spermatozoa to function properly, they must have smooth nuclei with normal chromatin content and normal head shape. Evaluation of semen parameters through semen analysis is therefore essential. Thereafter, based on findings, targeted interventions, including antioxidant supplementation, lifestyle modification, and medical interventions, can be applied to boost sperm quality and overall fertility potential. Quality intracytoplasmic morphologically selected sperm injection (IMSI) under ultra-magnification before injection into the oocyte is a possible strategy to boost embryo quality and overall pregnancy outcome [39]. Some study findings also point out that daily supplementation with alpha-lipoic acid (ALA) improves sperm motility and DNA damage in the male partner of couples with a history of RPL and ameliorates sperm lipid peroxidation, leading to higher levels of DNA compaction through augmenting the sperm nuclear protamine content, decreasing the rate of spontaneous pregnancy loss, especially before 24 weeks [84]. Another widely discussed intervention is paternal or unrelated donor lymphocyte therapy [62], although largely on an experimental scale.

**Lifestyle modification:** counselling couples with RPL on adopting healthy lifestyles may improve their chances of achieving successful conceptions and carrying the pregnancies to term. As there is an implication of lifestyle factors in the aetiology of RPL, assistive medical advice on lifestyle modification is an optimal and achievable first step towards achieving a favourable outcome [39]. Recommendations include smoking cessation because of its possible negative impact on live birth and achieving a healthy BMI range through exercise and healthy dieting. Collaborative efforts between care providers are therefore a vital venture to ensure compliance with these lifestyle modifications to improve the pregnancy outcomes.

## Discussion

It is widely acknowledged that RPL is a complex issue that arises from the interplay of numerous variables. This study provides a review of the potential aetiologies, focusing on immunological factors, parental chromosomal abnormalities, thrombophilias, uterine abnormalities, endocrinological factors, infections, male partner factors, maternal age, obesity, and lifestyle factors. Evidently, there have been considerable attempts at unpacking the aetiology of RPL and its subsequent diagnosis and management, although most of these studies are from upper-middle-income and high-income countries, with negligible advances from the African context, especially the sub-Saharan region. Previous studies have established this evidence gap between high- and low-income countries [95,96]. The dearth of evidence on RPL (RPL) in low- and middle-income countries (LMICs) could largely be attributable to underreporting and underdiagnosis of pregnancy losses, which are exacerbated by cultural and social factors. These factors contribute to the stigmatization of pregnancy loss, leading to insufficient documentation of cases.

Additionally, limited access to healthcare services, particularly in rural and remote areas, results in missed opportunities for diagnosis and management of RPL. This lack of accurate and comprehensive data on the incidence and prevalence of RPL poses a significant challenge in determining the actual burden of the condition and developing effective interventions. In this review, the burden of RPL is assessed primarily through prevalence and incidence measures reported in the included studies. Prevalence refers to the proportion of women within a defined population who experience RPL over a specified period, while incidence captures the rate of new RPL cases occurring during a defined time frame. Where available, additional indicators such as the number of pregnancy losses per woman and regional or country-level estimates were extracted to provide a more comprehensive understanding of the burden. These measures were synthesized quantitatively when feasible or narratively in cases of heterogeneous reporting.

Moreover, the root causes of RPL, including autoimmune diseases, are not well understood in several LMICs. Autoimmune diseases, such as antiphospholipid syndrome (APS), systemic lupus erythematosus (SLE), and thyroid autoimmunity, have been linked to RPL, but their occurrence and impact on pregnancy outcomes in LMICs are not well documented. The limited availability of diagnostic examinations, specialized healthcare providers, and immunomodulatory therapies further complicates efforts to detect and manage autoimmune-related RPL in LMIC settings. The absence of sufficient research and data collection impedes healthcare providers from recognizing and addressing autoimmune factors contributing to RPL, which results in subpar care for affected individuals and couples.

Recurrent pregnancy loss poses a considerable emotional and medical challenge for individuals and couples trying to start a family. Diagnosing RPL is a complex process that necessitates a meticulous evaluation of the medical history and potential contributing factors to miscarriages. Typically, the diagnostic process begins with a comprehensive medical history and a physical examination. This preliminary assessment aims to identify potential risk factors or underlying medical conditions, such as advanced maternal age, hormonal imbalances, structural abnormalities of the uterus, or genetic disorders, that may contribute to pregnancy loss. Next, laboratory tests are essential for detecting RPL. Blood tests can be performed to assess disorders in blood clotting, thyroid function, hormone levels, and autoimmune factors that may affect pregnancy outcomes [38,39,41,42,44-46,52,71,86,97]. Additionally, genetic tests, including parental karyotyping and chromosomal analysis of foetal tissue from earlier miscarriages, may also be recommended to identify chromosomal abnormalities that could increase the risk of pregnancy loss [39,40,42,46,59,65,67]. Further, imaging examinations such as hysterosalpingography or transvaginal ultrasound can also be conducted to assess the configuration of the uterus and identify any abnormalities, including uterine fibroids, polyps, or congenital defects, that might obstruct implantation or impede foetal development [39,41,44,46,77].

Diagnosis of RPL marks the start of a journey towards comprehending and tackling RPL. It is crucial to continuously monitor and offer support to guide treatment choices, track pregnancy results, and provide psychological support to individuals and couples grappling with the difficulties of RPL. Reproductive health issues RPL pose unique challenges in LMICs. Certain instances may necessitate specialized testing to investigate uncommon reasons for RPL. This may involve performing an endometrial biopsy to evaluate the uterine lining's receptivity, conducting immunological tests to assess immune system function and potential autoimmune influences, or carrying out thrombophilia screenings to gauge the likelihood of developing blood clotting disorders [38,39,41,42,46,47,50,51,54,55,57,58,64,74,88-92]. Further to the medical and laboratory evaluation, it is crucial to consider the emotional impact that RPL can have on both individuals and couples. The treatment approaches for RPL frequently depend on scant contextual evidence and can differ significantly based on local practices and available resources. Although lifestyle adjustments, supportive care, and empiric treatments, such as folic acid supplementation and progesterone therapy, may be recommended, their effectiveness in LMIC settings is not well established. Moreover, access to specialized care, such as assisted reproductive technologies (ART) and immunomodulatory therapies, is often restricted or unavailable in many LMICs, compounding the difficulties faced by individuals and couples dealing with RPL. Counselling and support services may be provided to assist individuals in dealing with the emotional stress of recurrent miscarriages and to offer guidance on coping mechanisms and family planning alternatives.

The cost implications of the diagnostic and treatment approaches, especially immunological and genetically oriented methods, in the cascade of management of RPL are considerable and often create barriers to accessing comprehensive care [46,47,67,69,73,74], especially in LMICs. For instance, although noted to be dropping in costs and being readily available, chromosomal microarray analysis (CMA) is estimated to cost above $1879.16 USD [44]. Therefore, only patients willing to accept the risk and cost of genetic chromosome screening will likely see a significant reduction in miscarriages and have favourable pregnancy outcomes [46]. In addition, many of these diagnostic tests are not fully covered by insurance, and out-of-pocket expenses can quickly pile up. Additionally, couples may require multiple tests and follow-up procedures, further increasing the financial burden. Hormonal discrepancies, including those involving thyroid hormones or progesterone levels, are frequently associated with RPL. Diagnostic approaches in this area include thyroid function tests, progesterone level assessments, and glucose tolerance tests to evaluate insulin resistance. Progesterone tests and thyroid function tests are commonly administered and cost-effective, typically priced between $100 and $200 per test. However, additional tests, such as glucose tolerance tests or advanced hormonal panels, may increase the overall expense. For couples experiencing RPL, these costs can accumulate as multiple tests may be necessary to reach a definitive diagnosis. To address these cost implications, healthcare providers must work towards more comprehensive insurance coverage for RPL-related diagnostics and offer flexible payment options for couples. Additionally, emphasis on preventive care and early detection can reduce long-term costs by enabling earlier interventions that improve pregnancy outcomes. As newer approaches become less costly and more widely available, they should undergo rigorous evaluation before being widely adopted. Evaluation of both efficacy and cost-effectiveness should be a research priority [67].

## Conclusion

Given the complexity and intricate nature of RPL, a multidisciplinary approach that includes specialists in reproductive endocrinology, maternal-foetal medicine, genetics, and psychology may prove to be beneficial in the management of RPL. Collaboration is essential in creating a comprehensive evaluation and personalized treatment plan for each patient. This is particularly important in low-income settings, where managing RPL requires early diagnosis, affordable treatment options, infection prevention, and emotional support. Therefore, cooperation between healthcare providers, research institutions, community programs, and external funding sources is crucial in promoting the integration of low- and middle-income countries (LMICs) into global research agendas and improving outcomes for couples experiencing RPL. Investing in research infrastructure, capacity building, and data collection systems is critical in generating high-quality evidence on the burden, causes, and treatment approaches of RPL in LMIC settings. Large, multinational, prospective studies are needed to evaluate the effectiveness of diagnostic tests in improving live-birth rates in women with RPL. Additionally, health economic studies are required to determine the cost-effectiveness of various tests in different healthcare settings. By prioritizing research on RPL and autoimmune diseases in LMICs, we can enhance our understanding of these complex conditions and develop evidence-based strategies to support individuals and couples affected by RPL in these settings.

### What is known about this topic

- Recurrent pregnancy loss is a multifactorial condition with established risk factors including chromosomal abnormalities, uterine anomalies, and thrombophilias;
- Despite advancements in diagnostic tools, more than 60% of RPL cases remain idiopathic, highlighting major gaps in understanding their pathophysiology.

### What this study adds

- There are evident disparities in access to advanced diagnostic and therapeutic technologies for RPL in LMICs compared to high-income countries;
- This study identifies tailored, resource-sensitive strategies as crucial for addressing RPL in LMICs, incorporating both clinical and sociocultural interventions to improve outcomes.
